# Topological triple-vortex lattice stabilized by mixed frustration in expanded honeycomb Kitaev-Heisenberg model

**DOI:** 10.1038/srep26750

**Published:** 2016-05-27

**Authors:** Xiaoyan Yao, Shuai Dong

**Affiliations:** 1 Department of Physics, Southeast University, Nanjing 211189, China

## Abstract

The expanded classical Kitaev-Heisenberg model on a honeycomb lattice is investigated with the next-nearest-neighboring Heisenberg interaction considered. The simulation shows a rich phase diagram with periodic behavior in a wide parameter range. Beside the double 120° ordered phase, an inhomogeneous phase is uncovered to exhibit a topological triple-vortex lattice, corresponding to the hexagonal domain structure of vector chirality, which is stabilized by the mixed frustration of two sources: the geometrical frustration arising from the lattice structure as well as the frustration from the Kitaev couplings.

The Kitaev model with the unconventional bond-dependent Kitaev interactions on the honeycomb lattice provided the first exactly solvable model with a quantum spin liquid ground state in two dimensions^1^. It is much highlighted since the Kitaev coupling was found to be realized in the honeycomb iridates which attracted considerable attention because they were suggested as the promising candidate materials for the topological band insulators^2^,^3^,^4^. Subsequently, the pure Kitaev model was generalized to a Kitaev-Heisenberg (KH) model with both the isotropic Heisenberg interaction and anisotropic Kitaev interaction on the nearest-neighboring sites, which was proposed to capture the magnetic interactions of honeycomb iridates^5^,^6^,^7^. However, a big challenge arose soon, that is, the magnetic orders observed in experiments were unexpected in the original KH model, such as the zigzag magnetic order experimentally observed in Na_2_IrO_3_^8^,^9^,^10^,^11^, and the incommensurate spiral magnetic state exhibited in the experiments of Li_2_IrO_3_^12^,^13^. While much effort was devoted to improve the original KH model to coincide with experimental results^13^,^14^,^15^,^16^,^17^,^18^,^19^,^20^, the exotic magnetic states existing in its various expanded forms have become another interesting topic. It is well-known that the frustration has long served as a relatively simple yet rich source of novel magnetic phases and exotic phenomena. In KH model, the unconventional Kitaev interaction breaks the spin rotational symmetry and provides an avenue for a new kind of frustration, namely the Kitaev frustration, which produces the unconventional zigzag and stripy states. As the geometrical frustration is introduced, new regions with puzzling magnetic states emerge on the phase diagram^14^,^18^, remaining unclear up to now. Recently Z_2_ vortex lattice excited by Kitaev-type anisotropic couplings in the triangular KH model was reported^21^,^22^, and it is expected that the similar topological state may exist in honeycomb KH model. Hitherto, to our knowledge, this interesting topic remains unsolved.

In the present paper, the expanded KH model on the honeycomb lattice is investigated by simulation. When the next-nearest-neighboring Heisenberg interaction is considered, the introduced geometrical frustration competing with the frustration arising from the anisotropic Kitaev interaction produces a rich phase diagram with periodic behavior on a wide parameter range. Beside the double 120° ordered phase where both triangular sublattices show 120° spin structure, a particular inhomogeneous phase with topological nontrivial modulation is stabilized by the mixed frustration of two sources. Extensive analysis reveals that different from the single Z_2_-vortex lattice observed in the triangular KH model^21^,^22^, this novel phase demonstrates a lattice of triplet topological Z_2_-vortexes on the triangular sublattice, which corresponds to a hexagonal domain structure of vector chirality.

## Results

**Figure 1:**
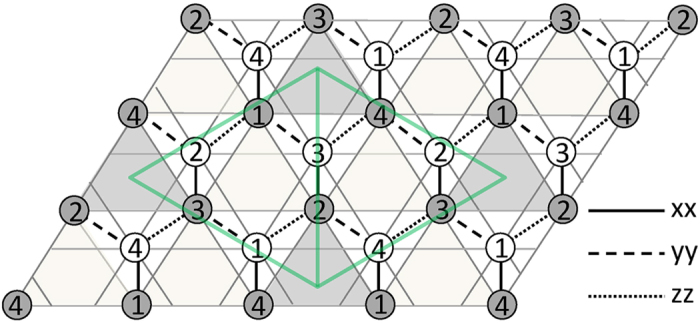
A sketch of honeycomb lattice, which is composed of two triangular sublattices as represented by white and gray dots respectively. The solid, dashed and dotted black thick lines indicate three kinds of spin-dependent nearest-neighboring bonds, where *xx*, *yy* and *zz* involve *S*^*x*^, *S*^*y*^, and *S*^*z*^ respectively. The gray thin lines present the links between the next nearest neighbors. Sites 1–4 specify the four sites for the spin rotations *S* → 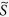. The shading illustrates the upward-pointing elementary triangles of one triangular sublattice, on which the vector chirality (*κ*) is calculated. Dark shading denotes every third upward-pointing triangles, and the vorticity (*v*) is evaluated on right- and left-pointing triangular loops (green) connecting these darker triangles.

**Figure 2:**
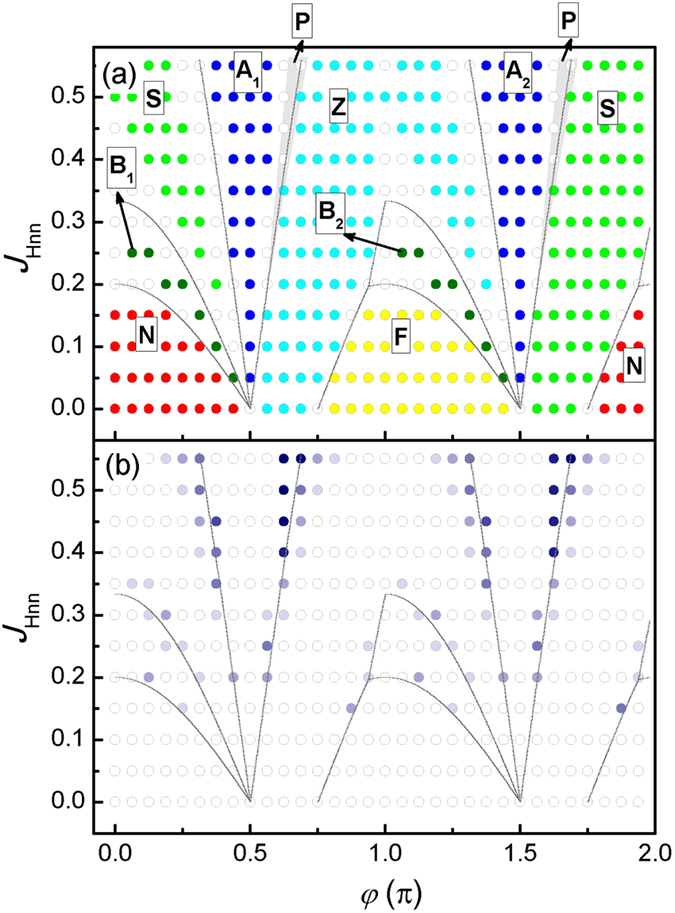
(**a**) The phase diagram, where the gray curves give the phase borders calculated from classic energy comparison. The solid circles filled with different colors show different phases identified by correlations obtained from simulation, and those empty circles represent the unidentified regions. The light gray regions schematically present the area of P phase. (**b**) The diagram of the fluctuation of local energy (*FLE*), where the shading of filled-circles refers to the value of *FLE* with white representing the minimum *FLE* = 0.

Considering the Kitaev interaction *J*_*Kn*_ coupling different spin components (*S*^*x*^, *S*^*y*^, and *S*^*z*^) on the nearest-neighboring bonds along the three lattice directions labeled by *γ* = *xx*, *yy* and *zz* as presented in Fig. 1, together with the isotropic Heisenberg couplings *J*_*Hn*_ and *J*_*Hnn*_ between spins on the nearest-neighboring (<i, j>) and the next-nearest-neighboring (≪i, k≫) sites, the Hamiltonian takes the form of,
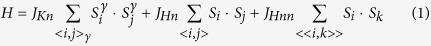
where *S*_*i*_ represents a classic spin with unit magnitude at the site *i*. *J*_*Hnn*_ takes positive value to introduce the geometrical frustration. The ratio of *J*_*Hn*_ to *J*_*Kn*_ is considered to its whole range by parameterizing *J*_*Hn*_ = cos*φ* and *J*_*Kn*_ = sin*φ* with *φ*: 0*~*2*π*. The simulation (see Methods) presents the phase diagram in the space of *φ* and *J*_*Hnn*_ as plotted in Fig. 2(a). Eight commensurate ordered phases can be detected by the standard values of correlations as listed in Table 1. The gray curves show the phase boundaries obtained by comparing the classic energies of these ordered phases, while the solid circles filled with different colors show the phases identified by correlations obtained from simulation (see Methods), and those empty circles represent the unidentified regions. It is seen that the results from energy calculation and simulation coincide well with each other.

**Figure 3:**
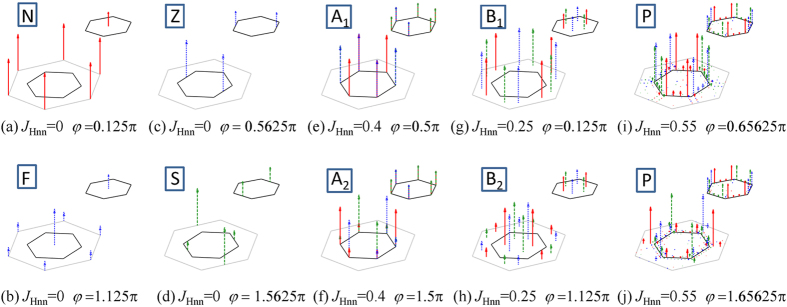
The typical spin structure factors for (**a**) N, (**b**) F, (**c**) Z, (**d**) S, (**e**) A_1_, (**f**) A_2_, (**g**) B_1_, (**h**) B_2_, (**i**,**j**) P phases. The main figure presents the spin structure factor on the honeycomb lattice (*Sh*^*γ*^(*k*)) where the gray large hexagon represents the extended Brillouin zone and the inner black hexagon indicates the first Brillouin zone. The small inset in the top right corner of each figure shows the spin structure factor on one triangular sublattice (*St*^*γ*^(*k*)). The solid (red), dashed (green) and dotted (blue) lines with arrows denote the signals from *S*^*x*^, *S*^*y*^ and *S*^*z*^ respectively.

It is well known that the honeycomb model with only nonzero *J*_*Hn*_ is unfrustrated, presenting the ferromagnetic (F) and Neel (N) orders as the ground states for *J*_*Hn*_ < 0 or *J*_*Hn*_ > 0. When *J*_*Kn*_ is switched on, the competition between *J*_*Hn*_ and *J*_*Kn*_ generates two nonconventional collinear orders: Stripy (S) and Zigzag (Z) phases. As displayed in Fig. 3(a–d), the spin structure factor on the honeycomb lattice (*Sh*^*γ*^(*k*), see Methods) for these four collinear phases shows very typical peaks, consistent well with previous reports^23^,^24^. It should be mentioned that these collinear orders could chose any one of *x*, *y* and *z* orientations due to the symmetry of the model, and thus the peaks of *Sh*^*γ*^(*k*) could show any one of three spin components with its locked bond direction. Moreover, it is noteworthy that F and N (S and Z) show the same spin structure for one triangular sublattice, and so the spin structure factor on one triangular sublattice (*St*^*γ*^(*k*), see Methods) exhibits the same feature. When *J*_*Hnn*_ is switched on, four interesting noncollinear phases (A_1_, A_2_, B_1_ and B_2_) with the typical correlation values emerge from the Kitaev points of *φ* = 0.5π and 1.5π. A_1_ and A_2_ phases locate around the vertical lines at *φ* = 0.5π and 1.5π, spreading with *J*_*Hnn*_increasing. As plotted in Fig. 3(e,f), *Sh*^*γ*^(*k*) presents the sharp peaks at the corners of the first Brillouin zone (K points) for both A_1_ and A_2_ phases. Although a detailed difference exists on *Sh*^*γ*^(*k*), *St*^*γ*^(*k*) exhibits the same feature for A_1_ and A_2_, which means the coplanar 120° spin order on each triangular sublattice where every two neighboring spins form an angle of 120°. Moreover, in such a double 120° phase, the 120° spin structures on two triangular sublattices are also coplanar and lie in one of the {111} planes^14^,^20^. Different from A_1_ and A_2_ phases, B_1_ and B_2_ exist only with weak *J*_*Hnn*_. Although they show very different peaks of *Sh*^*γ*^(*k*) as plotted in Fig. 3(g,h), their *St*^*γ*^(*k*) gives the same feature with the peaks on the points along the Γ-K line (Γ is the center of first Brillouin zone). It is interesting that B_1_ and B_2_ can be transformed into A_1_ and A_2_ respectively by a four-site transformation *S* → 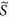
5,25,26, namely
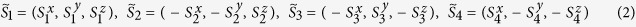
where the four sites are labeled as Fig. 1. It should be mentioned that the four-site transformation of Eq. (2) implies the Klein duality^22^,^27^, which strictly holds for the nearest-neighboring KH model with the mapping relation: *J*_*Hn*_ to –*J*_*Hn*_ and *J*_*Kn*_ to 2*J*_*Hn*_+*J*_*Kn*_. The duality could also exist in the model augmented with weak *J*_*Hnn*_ to some extent, but the original mapping relation of coupling parameters is complicated by *J*_*Hnn*_. Since both *Sh*^*γ*^(*k*) and *St*^*γ*^(*k*) calculated on 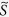 of B_1_ and B_2_ show the same feature to A_1_ and A_2_ respectively, B_1_ and B_2_ can be regarded as the Klein dual phases of A_1_ and A_2_.

Between the commensurate phases mentioned above, there are unidentified regions about the phase boundaries, which are puzzling and could be more attractive. The fluctuation of local energy (*FLE,* see Methods) just presents nonzero value around these intermediate regions as displayed in Fig. 2(b). It is noteworthy that *FLE* exhibits a period of π on *φ*, which corresponds to the periodic behavior of spin configuration on the triangular sublattice. As illustrated in Fig. 3, every two states with *φ* difference of π have the same *St*^*γ*^(*k*), indicating the same magnetic structure on the triangular sublattice. If the phase diagram is marked by the spin states on triangular sublattice, that is, FN includes F and N, SZ includes S and Z, A includes A_1_ and A_2_, B includes B_1_ and B_2_, the obtained phase diagram shows a perfect period of π. The *φ* difference of π means the change of sign for both *J*_*Hn*_ and *J*_*Kn*_, and then this periodic behavior on phase diagram just results from the energy symmetry of the bipartite honeycomb lattice^28^, which is well retained even with the geometrical frustration introduced by *J*_*Hnn*_. To avoid repeat, only the range of *φ:* π*~*2π is discussed blow, which includes the region of *J*_*Hn*_ > 0 and *J*_*Kn*_ *<* 0 as originally proposed for the KH model relevant to iridates^2^,^5^. The nonzero *FLE* corresponds to the inhomogeneous magnetic structure, appearing around the double 120° (A) and its Klein dual (B) phases. Since B phase can be mapped onto A, we only focus on the areas beside A, which are wider and have higher *FLE* value than the narrow regions around B.

**Figure 4:**
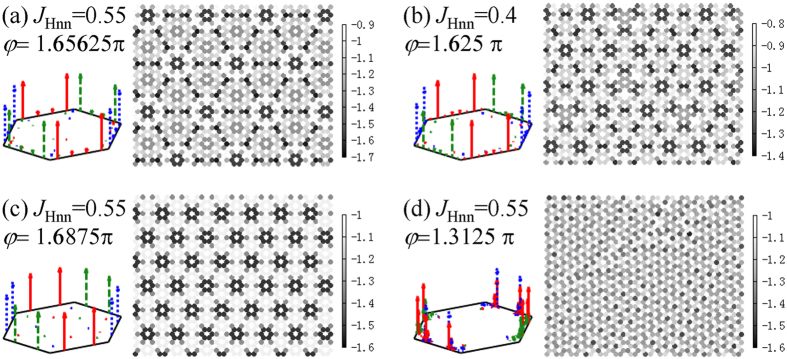
The spin structure factor on the triangular sublattice (*St*^*γ*^(*k*)) is shown on the left of each figure for the states (**a**–**c**) in P phase and (**d**) in the region on the left of A phase. The solid (red), dashed (green) and dotted (blue) lines with arrows denote the signals from *S*^*x*^, *S*^*y*^ and *S*^*z*^ respectively. Correspondingly, the maps of *E*_*i*_ on the honeycomb lattice are given on the right. For visibility, only part of the lattice is shown here, and the shading refers to the value of *E*_*i*_.

In the inhomogeneous region on the right of A, a particular ordered (P) phase is found to show *St*^*γ*^(*k*) of sharp peaks separately for three spin components and *E*_*i*_ map with very clear pattern, implying a nontrivial modulation on the magnetic structure. When *φ* is changed from A to SZ phase, the main peaks of *St*^*γ*^(*k*) slide along the hexagon’s edges from K to M points (M is the midpoint of every edge)^14^, that is, if the wave vector of main peak is denoted by *k*_*p*_, its magnitude ^|^*k*_*p*_| changes from 1.33333 (K point) to 1.15470 (M point). Fig. 4(a–c) displays three states in this phase with different |*k*_*p*_|, namely, (a) the state at *J*_*Hnn*_ = 0.55 and *φ* *=* 1.65625π with |*k*_*p*_| = 1.25830, (b) the state at *J*_*Hnn*_ = 0.4 and *φ* *=* 1.625π with |*k*_*p*_| = 1.22758, and (c) the state at *J*_*Hnn*_ = 0.55 and *φ* *=* 1.6875π with |*k*_*p*_| = 1.20185. Here (b) state just shows the value of |*k*_*p*_| between those of (a) and (c). The modulation becomes denser with |*k*_*p*_| decreasing, which could be induced by increasing *φ* or reducing *J*_*Hnn*_. P phase exists in the narrow region on the right of A phase along the boundary as illustrated in Fig. 2(a), spreading and moving to higher *φ* when *J*_*Hnn*_ is raised. In contrast, *St*^*γ*^(*k*) in the region on the left of A shows the peaks with different spin components mixed, and no obvious texture can be observed in the map of *E*_i_ (Fig. 4(d)).

## Discussion

**Figure 5:**
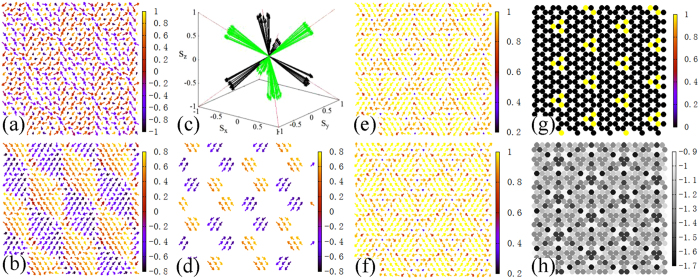
The state of *J*_*Hnn*_ = 0.55 and *φ* = 1.65625π. (**a**) Spin configuration on one triangular sublattice, where the arrows show the projections of spins onto *x-y* plane with the color denoting the value of *S*^*z*^. (**b**) *κ*(*r*) configuration on one triangular sublattice, where the arrows present the projections of *κ* onto *x-y* plane and the color refers to *κ*^*z*^. (**c**) Configuration of *κ* vectors with |*κ*| > 0.996 and their ends moved to zero. The black arrows represent *κ* from one triangular sublattice and the green arrows from another one. (**d**) *κ*(*r*) configuration on one triangular sublattice with |*κ*| > 0.996 and the color representing the value of *κ*^*z*^. (**e**,**f**) *κ*(*r*) configurations on two triangular sublattices with the color referring to the value of |*κ*|. (**g**) Map of vorticity (*v*) with the color denoting the value of *v*. (**h**) Map of *E*_*i*_ on one triangular sublattice which is extracted from the *E*_*i*_ map in Fig. 4(a). For visibility, only part of the lattice is shown here.

In order to understand this nontrivial modulation, the magnetic states in P phase are discussed in detail. The texture of *E*_i_ map may correspond to the defect on magnetic structure, which is hard to be observed directly on the spin configuration of honeycomb lattice. When one triangular sublattice is extracted, the slightly distorted 120° spin structure can be found on most elementary triangles as displayed in Fig. 5(a). In this case, an elementary ordering unit consists of three spins on a triangle, which can be evaluated by the chirality vector (*κ*, see Methods). The averaged length of *κ* gives <|*κ*|> = 0.897, 0.862 and 0.832 respectively for the states in Fig. 4(a–c), indicating that 120° structure is kept locally in this phase.

At the state with *J*_*Hnn*_ = 0.55 and *φ* = 1.65625π, the *κ*(*r*) configuration calculated on one triangular sublattice exhibits a very regular pattern composed of different *κ* domains as plotted in Fig. 5(b). In order to observe the orientations of these *κ* domains, the main part of domains, namely the *κ* vectors with |*κ*| > 0.996 are extracted and plotted in one chart with their ends moved to zero. As plotted in Fig. 5(c), one triangular sublattice will choose four of eight <111> directions, which just point to the four corners of a tetrahedron. And the other triangular sublattice will choose the other four directions. Note that A phase has the perfect 120° structure lying in one of the {111} planes, namely its *κ* points to one of eight <111> directions. Actually P is a phase with the coexistence of *κ* domains in all these eight directions. If Fig. 5(b) is replotted by hiding the *κ* vectors with |*κ*| < 0.996, a beautiful pattern can be found (Fig. 5(d)), which clearly shows that the *κ* domains with four orientations constitute four honeycomb lattices intersecting with each other. The domain walls are constructed between these *κ* domains, which are clearer when the shading marks the magnitude of *κ*, i.e. |*κ*|. As displayed in Fig. 5(e), the domain walls with smaller |*κ*| form a regular lattice, surrounding the triangular domains of *κ*. It is interesting that on the intersection of six domain walls, there are always three points with the smallest |*κ*| as the “vortex cores”, constituting a downward pointing triangle. In contrast, on the other triangular sublattice, the similar three “vortex cores” are placed on an upward pointing triangle as presented in Fig. 5(f). This *κ* vortex structure is confirmed by the map of vorticity (*v*, see Methods) in Fig. 5(g), where triplet vortexes with *v* = 1 just locate at the same place. Since it is close to A phase and 120° structure is kept locally on the triangular sublattice, the vortex here is Z_2_ type due to the order parameter space SO(3)^29^. Comparing to the *E*_*i*_ map of one triangular sublattice as plotted in Fig. 5(h), the domain walls and the vortex-triplets just correspond to the texture observed on *E*_*i*_ map.

**Figure 6:**
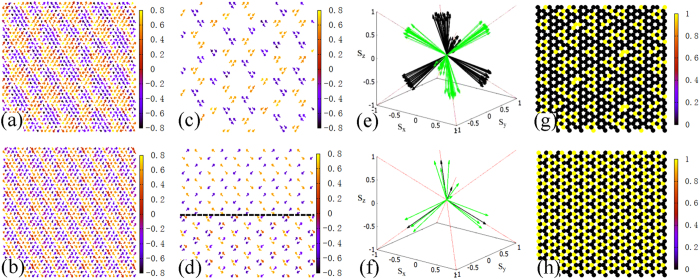
The upper line (**a**,**c**,**e**,**g**) shows the state of *J*_*Hnn*_ = 0.4 and *φ* = 1.625π. The lower line (**b**,**d**,**f**,**h**) presents the state of *J*_*Hnn*_ = 0.55 and *φ* = 1.6875π. The first column (**a**,**b**) gives *κ*(*r*) configurations on one triangular sublattice. The second column presents *κ*(*r*) configurations (**c**) with |*κ*| > 0.98 on one triangular sublattice and (**d**) with |*κ*| > 0.92 on two triangular sublattices separated by a dashed line. The arrows show the projections of *κ* onto *x-y* plane with the color referring to the value of *κ*^*z*^. The third column displays the configurations of *κ* vectors with their ends moved to zero in the case of (**e**) |*κ*| > 0.98 and (**f**) |*κ*| > 0.92. The black arrows represent *κ* from one triangular sublattice and the green arrows from another one. The fourth column (**g**,**h**) gives the maps of vorticity (*v*) with the color denoting the value of *v*. For visibility, only part of the lattice is shown here.

The increase of |*k*_*p*_| corresponds to a denser modulation, which can be seen on *κ*(*r*) configurations as displayed in Fig. 6(a,b). In the case of *J*_*Hnn*_ = 0.4 and *φ* = 1.625π, although *κ* domains shrink, they still exist and form a regular pattern, which can be seen clearly by hiding the *κ* vectors with |*κ*| < 0.98 (Fig. 6(c)). The orientations of these *κ* domains still keep to the four corners of one tetrahedron for one sublattice, and the other sublattice chooses the remaining four directions, as shown in Fig. 6(e). When *J*_*Hnn*_ = 0.55 and *φ* = 1.6875π, the modulation becomes too dense to distinguish different domains. Whatever, by hiding the *κ* vectors with |*κ*| < 0.92, the framework of *κ*(*r*) configuration can be found as displayed in Fig. 6(d). Different from the aforementioned two cases with longer |*k*_*p*_| where *κ*(*r*) configurations show similar structures on two triangular sublattices, in this case different patterns are found. Moreover, the orientations of these dominant *κ* on two triangular sublattices tend to fall to the four corners of one tetrahedron, as shown in Fig. 6(f). The maps of vorticity in Fig. 6(g,h) also demonstrate denser texture with shorter |*k*_*p*_|, but the vortex-triplets can still be discerned.

It is noteworthy that this phase with topological triple-vortex lattice results from the mixed frustration of geometrical and Kitaev sources. *J*_*Kn*_, *J*_*Hn*_ and *J*_*Hnn*_ are all important to produce such a phase. If *J*_*Hnn*_ = 0, namely the geometrical frustration is switched off as the horizontal bottom, the Kitaev frustration produces only homogenous states even at the spin liquid points. If *J*_*Kn*_ = 0, namely the Kitaev frustration is turned off as the upright lines at *φ* = 0 and π, the geometrical frustration only generates homogenous FN and spiral states^30^,^31^. If *J*_*Hn*_ = 0 as the upright lines at *φ* = 0.5π and 1.5π, there is only A phase also with *FLE* = 0. Furthermore, although inhomogeneous states may exist in the region on the left of A phase, the same signs of *J*_*Hn*_ and *J*_*Kn*_ can not enable an effective Kitaev frustration, and thus the vortex lattice can not be observed.

In summary, the honeycomb KH model expanded by considering the next-nearest-neighboring Heisenberg interaction is investigated in a wide parameter space. The simulation shows a rich phase diagram with periodic behavior. Besides the well-known F, N, S and Z orders, the noncollinear A and B phases are identified as the double 120° and its Klein dual phases. On the right of A, an inhomogeneous phase with nontrivial modulation is uncovered, which is stabilized by the mixed frustration of geometrical and Kitaev sources, and persists at temperature T → 0. Further analysis reveals that this is a particular ordered phase corresponding to the hexagonal domain structure of vector chirality, and on the crossings of these domain walls the topological Z_2_-vortex triplets form a lattice. This novel magnetic phase is stable in a parameter region relevant to iridates, since further-neighboring interaction beyond the nearest-neighboring one has been strongly suggested to explain the experimental results^10^,^18^.

## Methods

The simulation is performed on the Hamiltonian of Eq. 1. Fixing
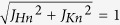
 as the energy scale, the ratio of *J*_*Hn*_ to *J*_*Kn*_ is considered to its whole range by parameterizing *J*_*Hn*_ = cos*φ* and *J*_*Kn*_ = sin*φ* with *φ*: 0*~*2π. The Monte Carlo simulation of the Metropolis algorithm combined with the over-relaxation method is performed on the honeycomb lattice of N = 9216 sites with periodic boundary conditions assumed^32^,^33^, and one unit Monte Carlo step (MCS) consists of ten over-relaxation sweeps and one Metropolis sweep. On every parameter point, the system is fully relaxed and evolved by a gradual cooling procedure from a random initial state to a very low temperature T = 0.0000002 (6000 MCSs at every one of 20 intermediate temperatures). Then the energy is further minimized by 50000 MCSs restricted at T = 0 (namely only the proposed update with the energy variation not higher than zero is accepted) to approach the limit of zero temperature. The final result is obtained by comparing more than 10 independent data sets evolving from different initial states.

The obtained states are robust at the corresponding parameter points, which exist stably at low temperature, and survive even with perturbation from disorder or anisotropy. Based on the state obtained, the correlations on the nearest-neighboring spin pairs (*C*_n_), on the next-nearest-neighboring pairs (*C*_nn_), on the third-nearest-neighboring pairs (*C*_nnn_) and on the nearest-neighboring Kitaev bonds are calculated by
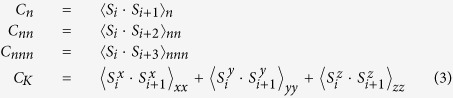


Eight commensurate ordered states can be identified by the standard values of correlations listed in Table 1 with less than 15 percent deviation. To further confirm or distinguish the magnetic states, the spin structure factor on the honeycomb lattice (*Sh*^*γ*^(*k*)) is calculated in the form of 23
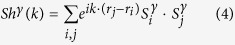


In addition, since the honeycomb lattice is composed of two triangular sublattices, the spin structure factor can also be calculated on one triangular sublattice (*St*^*γ*^(*k*)), namely,
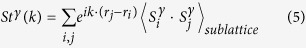


In both cases, the spin structure factors are calculated for three spin components (*γ* = *x*, *y* and *z*) respectively to show the detailed spin structure induced by the anisotropic Kitaev interaction.

To locate the inhomogeneous states, the fluctuation of local energy (*FLE*) is evaluated on the whole honeycomb lattice in the form of
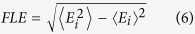


The site-dependent local energy (*E*_i_) is calculated by
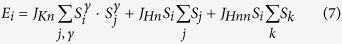
where *j* denotes the nearest-neighboring bonds and *k* denotes the next-nearest-neighboring bonds. The map of *E*_i_ can be checked to detect the nontrivial modulation on magnetic structure. To further analyze the nontrivial spin modulation, the chirality vector (*κ*) is calculated on the spin configuration of one triangular sublattice in the form of^29^,^34^
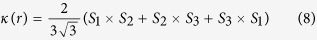
where the corner sites 1, 2 and 3 are numbered clockwise for every upward pointing elementary triangle (Fig. 1). The orientation of *κ* is perpendicular to the plane on which the 120° spin structure lies approximately. The length of *κ* (|*κ*|) gives a measure of the rigidity of 120° structure, and it is normalized to give unity for a perfect 120° structure. If the spin configuration keeps 120° structure locally, the vorticity (*v*) can be calculated in the same manner as refs 21 and 29 by going around the right- and left-pointing triangular loops as illustrated in Fig. 1. *v* is rescaled to be 0 for no rotation and 1 for a rotation by 2π.

## Additional Information

**How to cite this article**: Yao, X. and Dong, S. Topological triple-vortex lattice stabilized by mixed frustration in expanded honeycomb Kitaev-Heisenberg model. *Sci. Rep.* **6**, 26750; doi: 10.1038/srep26750 (2016).

## Figures and Tables

**Table 1:** The standard values of the correlations *C*_*n*_, *C*_*nn*_, *C*_*nnn*_ and *C*_*K*_ for F, N, S, Z, A_1_, A_2_, B_1_ and B_2_ phases.

	F	N	S	Z	A_1_	A_2_	B_1_	B_2_
*C*_*n*_	3	−3	−1	1	0	0	−2	2
*C*_*nn*_	6	6	−2	−2	−3	−3	1	1
*C*_*nnn*_	3	−3	3	−3	0	0	0	0
*C*_*K*_	1	−1	−1~1	−1~1	−1	1	−1	1
